# Decorrelation of the True and Estimated Classifier Errors in High-Dimensional Settings

**DOI:** 10.1155/2007/38473

**Published:** 2007-10-30

**Authors:** Blaise Hanczar, Jianping Hua, Edward R Dougherty

**Affiliations:** 1 Department of Electrical and Computer Engineering, Texas A&M University, College Station, TX 77843, USA; 2 Laboratoire d'Informatique Medicale et Bio-informatique (Lim&Bio), Universite Paris 13, Bobigny cedex 93017, France; 3 Computational Biology Division, Translational Genomics Research Institute, Phoenix, AZ 85004, USA

## Abstract

The aim of many microarray experiments is to build discriminatory diagnosis and prognosis models. Given the huge number of features and the small number of examples, model validity which refers to the precision of error estimation is a critical issue. Previous studies have addressed this issue via the deviation distribution (estimated error minus true error), in particular, the deterioration of cross-validation precision in high-dimensional settings where feature selection is used to mitigate the peaking phenomenon (overfitting). Because classifier design is based upon random samples, both the true and estimated errors are sample-dependent random variables, and one would expect a loss of precision if the estimated and true errors are not well correlated, so that natural questions arise as to the degree of correlation and the manner in which lack of correlation impacts error estimation. We demonstrate the effect of correlation on error precision via a decomposition of the variance of the deviation distribution, observe that the correlation is often severely decreased in high-dimensional settings, and show that the effect of high dimensionality on error estimation tends to result more from its decorrelating effects than from its impact on the variance of the estimated error. We consider the correlation between the true and estimated errors under different experimental conditions using both synthetic and real data, several feature-selection methods, different classification rules, and three error estimators commonly used (leave-one-out cross-validation, -fold cross-validation, and .632 bootstrap). Moreover, three scenarios are considered: (1) feature selection, (2) known-feature set, and (3) all features. Only the first is of practical interest; however, the other two are needed for comparison purposes. We will observe that the true and estimated errors tend to be much more correlated in the case of a known feature set than with either feature selection or using all features, with the better correlation between the latter two showing no general trend, but differing for different models.

## 

[1,2,3,4,5,6,7,8,9,10,11,12,13,14,15,16,17,18,19,20,21,22]
